# Supplementary material for: Age determines NK cell fate and tissue compartmentalization to CMV infection
Source: bioRxiv. 2026 Mar 17:2026.03.16.712099. Preprint. [Version 1] doi: 10.64898/2026.03.16.712099 (PMC13015448; doi:10.64898/2026.03.16.712099)
Supplement: Supplement 1 [file NIHPP2026.03.16.712099v1-supplement-1.pdf]

bioRxiv preprint doi: https://doi.org/10.64898/2026.03.16.712099; this version posted March 17, 2026. The copyright holder for this preprint (which was not certified by peer review) is the author/funder, who has granted bioRxiv a license to display the preprint in perpetuity. It is made available under aCC-BY 4.0 International license.

## SUPPLEMENTARY MATERIALS

Figure S1. Flow cytometry gating strategies.

Figure S2. NK cell kinetics over age.

Figure S3. Fine-grained clustering of NK cell transcriptional states.

Figure S4. Kinetics of the $CD8^+$ T cell response to MCMV infection in adult mice across sites.

Figure S5. Transcriptomic distinctions among infant and adult naive and MCMV-specific T cells in acute infection.

Figure S6. Memory and effector potential of MCMV-specific T cell populations.

Figure S7. Organ donor cohort demographics.

Figure S8. Age and tissue influence adaptive-like NK cell and $CD8^+$ $T_{EMRA}$ cell phenotypic composition.

Table S1. Adult and Infant NK cell differential gene expression at 0,7,15 DPI.

Table S2. Adult and Infant OT-I CD8 T cell differential gene expression at 0,7 DPI.

Table S3. Gene Rank of Adaptive-like NK cell scHPF signature.

Table S4. Donor Information Table

Table S5. Key resources used in this study.

bioRxiv preprint doi: https://doi.org/10.64898/2026.03.16.712099; this version posted March 17, 2026. The copyright holder for this preprint (which was not certified by peer review) is the author/funder, who has granted bioRxiv a license to display the preprint in perpetuity. It is made available under aCC-BY 4.0 International license.

# Supplementary Materials for

## Age determines NK cell fate and tissue compartmentalization to CMV infection

Isaac J. Jensen, Benjamin J. Redenti, Steven B. Wells, Joshua I. Gray, Daniel P. Caron, Donna L. Farber

Corresponding author: df2396@cumc.columbia.edu

**The PDF file includes:**

Figs. S1 to S8
Table S1 to S5

bioRxiv preprint doi: https://doi.org/10.64898/2026.03.16.712099; this version posted March 17, 2026. The copyright holder for this preprint (which was not certified by peer review) is the author/funder, who has granted bioRxiv a license to display the preprint in perpetuity. It is made available under aCC-BY 4.0 International license.

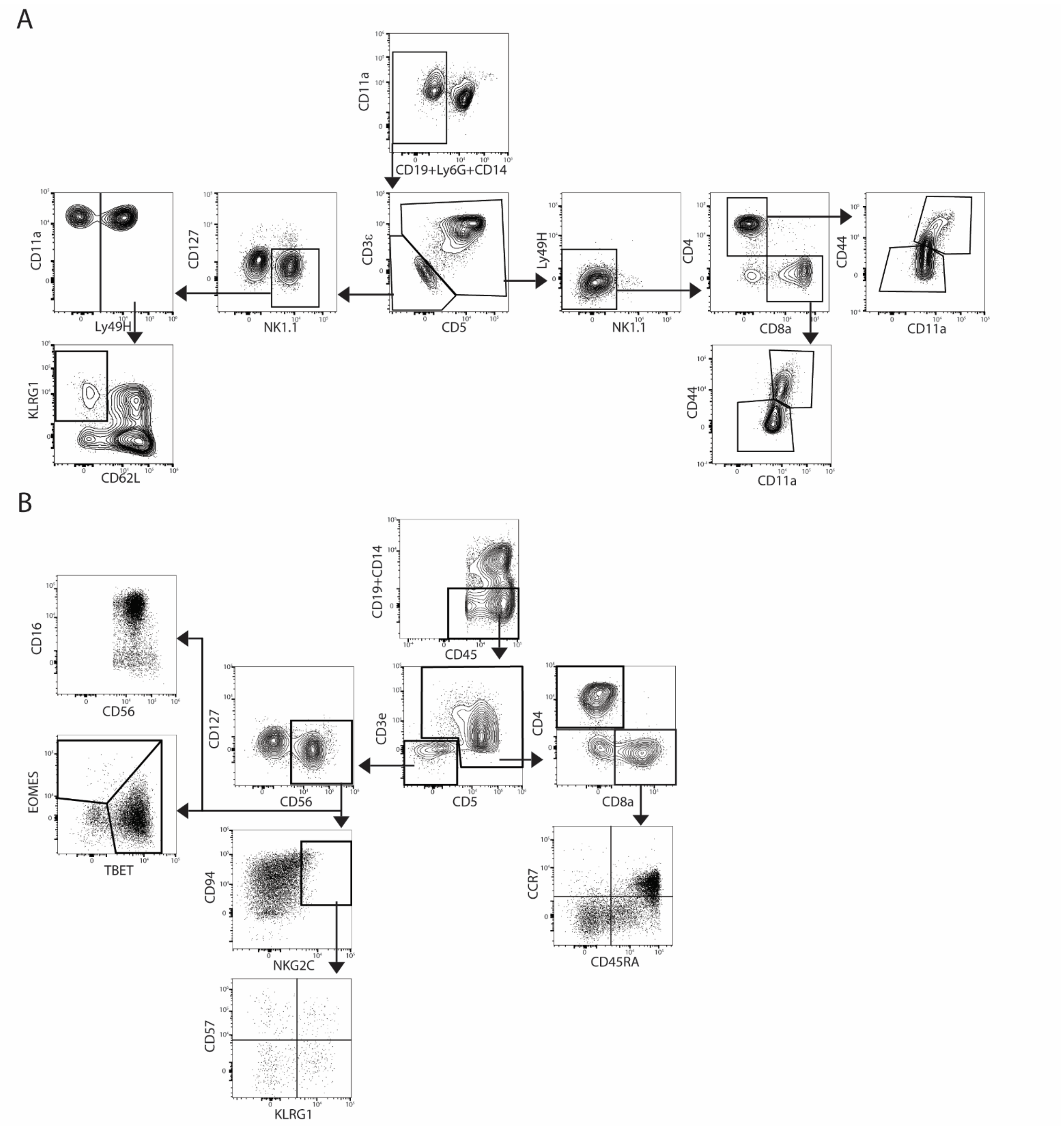


**Figure S1. Flow cytometry gating strategies.**
A) Mouse flow cytometry gating strategy for NK cells (CD3$^-$CD5$^-$CD19$^-$CD14$^-$Ly6G$^-$CD127$^-$NK1.1$^+$), identification of MCMV-reactive Ly49H$^+$ NK cells and adaptive-like (Ly49H$^+$KLRG1$^+$CD62L$^-$) NK cells, and T cells (CD3$^+$ CD5$^+$CD19$^-$Ly6G$^-$NK1.1$^-$) including CD8$^+$ T cells, CD4$^+$ T cells, antigen-experienced (CD44$^{hi}$CD11a$^{hi}$), and naïve (CD44$^{lo}$CD11a$^{lo}$) subsets. (B) Human flow cytometry gating strategy for NK cells (CD3e$^-$CD5$^-$CD19$^-$CD14$^-$CD127$^-$CD56$^+$), identification of immature NK cells (CD56$^{bright}$ CD16$^-$ or EOMES$^{hi}$ TBET$^{lo}$), mature NK cells (CD56$^{dim}$ CD16$^+$ or EOMES$^{lo}$ TBET$^{hi}$), HCMV-reactive (NKG2C$^+$CD94$^+$) NK cells, and adaptive-like (Ly49H$^+$CD94$^+$[KLRG1$^+$ and/or CD57$^+$]) NK cells, and T cells (CD3$^+$ CD5$^+$CD19$^-$CD14$^-$) including CD8+ T cells, naive ($T_N$; CD45RA$^+$ CCR7$^+$), central memory ($T_{CM}$; CD45RA$^-$ CCR7$^+$), effector memory ($T_{EM}$; CD45RA$^-$ CCR7$^-$), and effector memory expressing CD45RA ($T_{EMRA}$; CD45RA$^+$ CCR7$^-$) subsets.

bioRxiv preprint doi: https://doi.org/10.64898/2026.03.16.712099; this version posted March 17, 2026. The copyright holder for this preprint (which was not certified by peer review) is the author/funder, who has granted bioRxiv a license to display the preprint in perpetuity. It is made available under aCC-BY 4.0 International license.

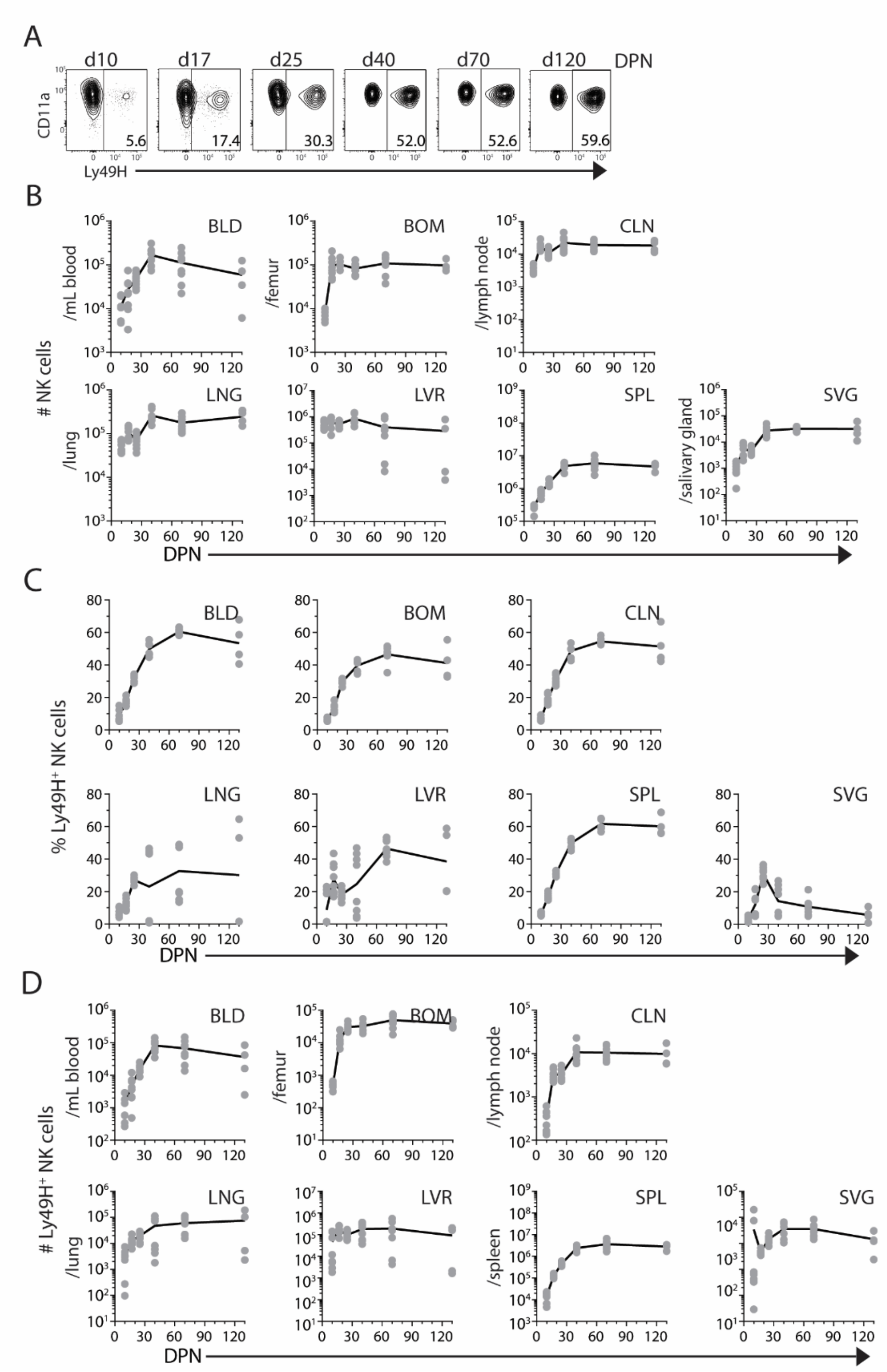


**Figure S2. NK cell kinetics over age.**
(A) Representative gating for splenic Ly49H$^+$ NK cells at indicated days post-natal (DPN). (B) Number of NK cells across tissues at indicated ages. (C) Frequency and (D) number of Ly49H$^+$ NK cells in tissues at indicated ages. n = 8 mice per time-point per group. Data are combined from N=2 individual experiments. Tissue Abbreviations: blood (BLD), bone marrow (BOM), cervical lymph node (CLN), lung (LNG), liver (LVR), spleen (SPL), and salivary gland (SVG)

bioRxiv preprint doi: https://doi.org/10.64898/2026.03.16.712099; this version posted March 17, 2026. The copyright holder for this preprint (which was not certified by peer review) is the author/funder, who has granted bioRxiv a license to display the preprint in perpetuity. It is made available under aCC-BY 4.0 International license.

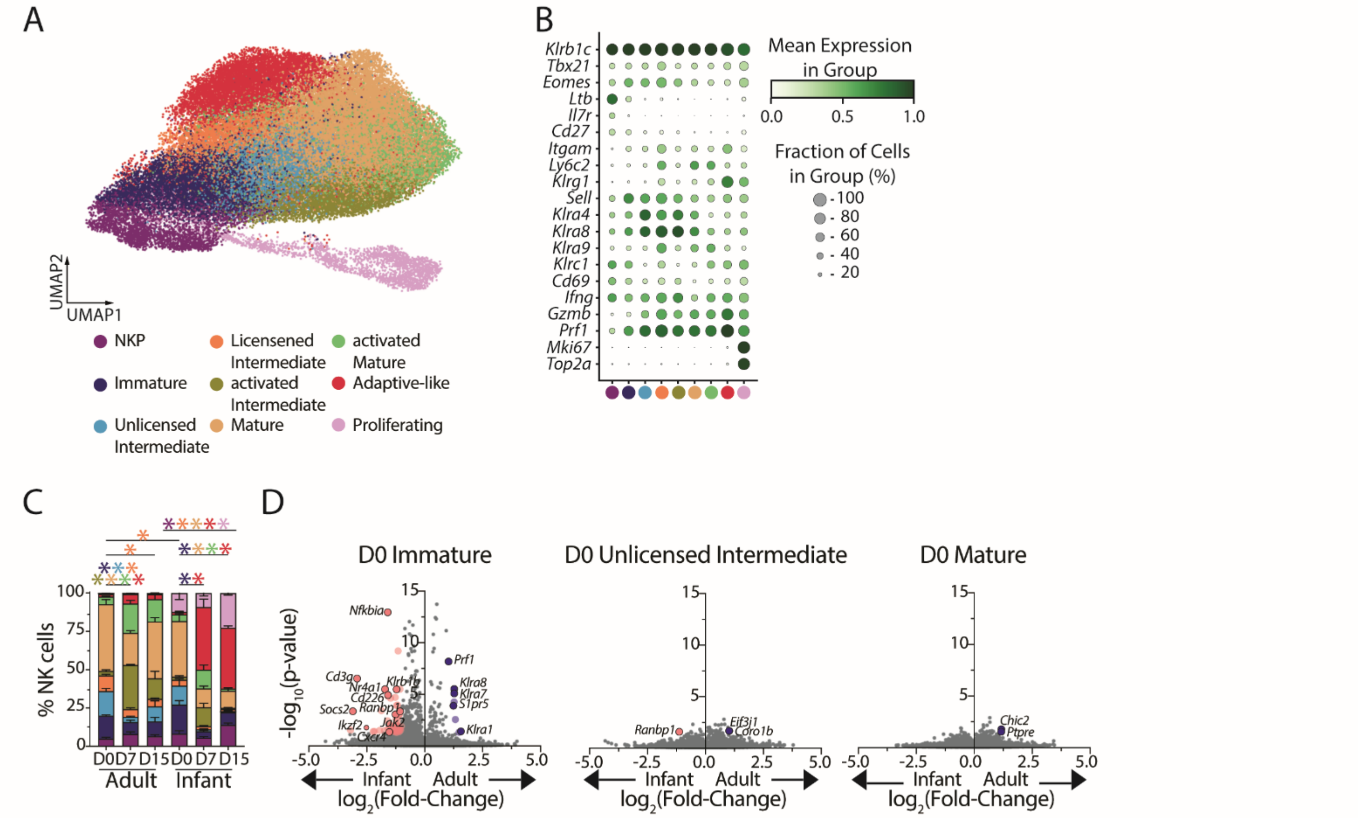


**Figure S3. Fine-grained clustering of NK cell transcriptional states.**

NK cells were isolated by magnetic enrichment from the spleens of naive and adult mice prior to infection (D0) and 7 and 15 days post-MCMV infection (D7, D15) for CITE-seq profiling using the 10x genomics system. (A) Leiden clustering of adult and infant NK cells pre- and post-infection, with legend indicating putative cluster identity. (B) Dot plot displaying the relative frequency and degree of expression for indicated genes that define subsets based on Leiden clusters in panel A. (C) Proportion of each NK cell subset for adult and infant mice pre- and post-infection. n = 4 recipients per age group per timepoint; *p<0.05 as assessed by two-way ANOVA with Bonferroni multiple comparisons test; asterisk color corresponds to the population that is significantly different. (D) Volcano plots displaying differentially expressed genes (pink/blue dots; p-value <0.05, $log_2$(fold change)>1) between adult and infant prior to infection among immature, unlicensed intermediate, and mature NK cells.

bioRxiv preprint doi: https://doi.org/10.64898/2026.03.16.712099; this version posted March 17, 2026. The copyright holder for this preprint (which was not certified by peer review) is the author/funder, who has granted bioRxiv a license to display the preprint in perpetuity. It is made available under aCC-BY 4.0 International license.

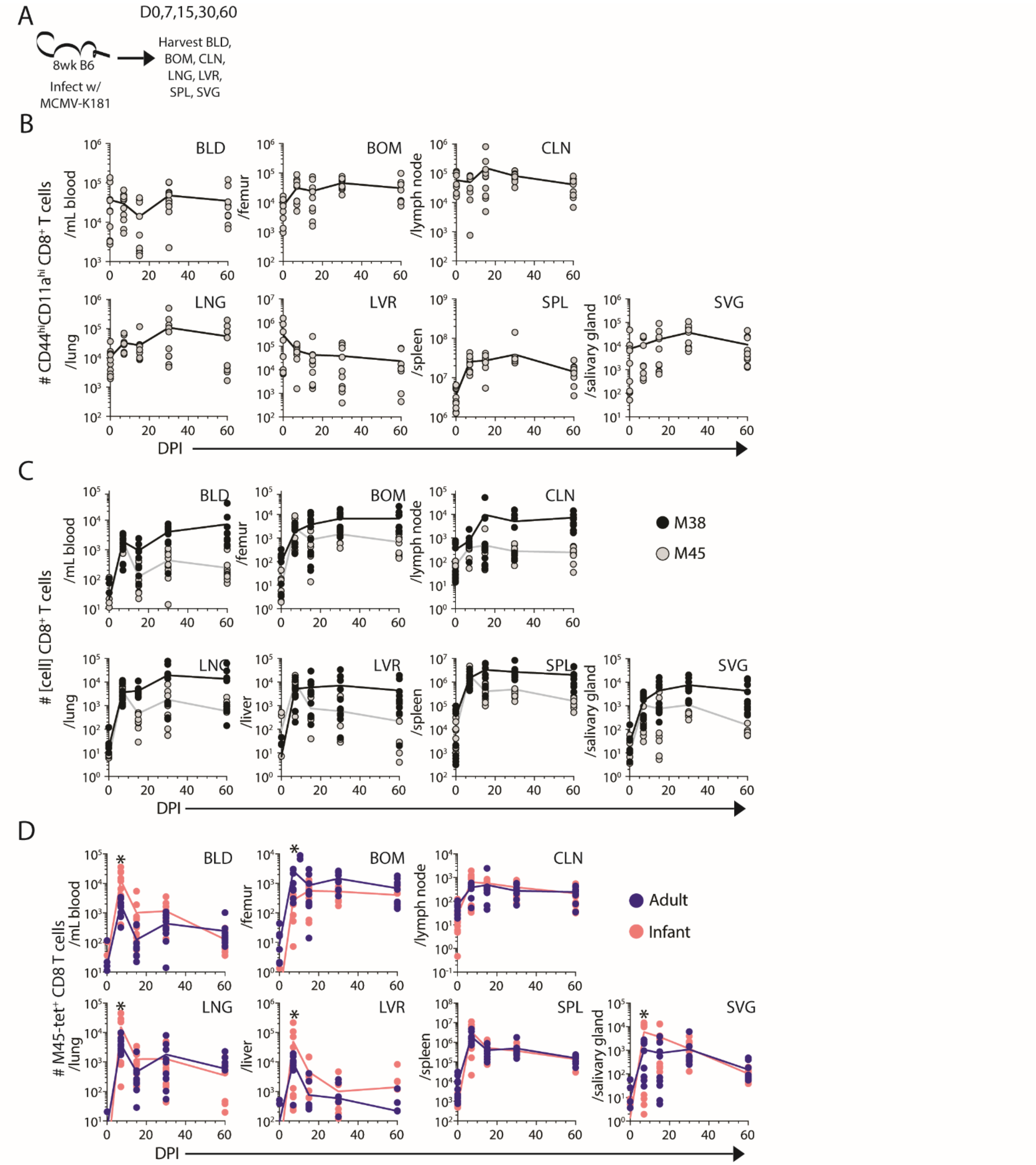


**Figure S4. Kinetics of the CD8$^+$ T cell response to MCMV infection in adult mice across sites.**
(A) Mice were infected with MCMV and blood and tissues were harvested at 0-60days post-infection. (B) Number of antigen-experienced (CD44$^{hi}$CD11a$^{hi}$) CD8 (circles) T cells per tissue site at indicated day post infection (DPI). (C) Number of MCMV-specific M38 (black circle) and M45 (grey circle) CD8$^+$T cells across tissues at indicated DPI. Number of (D) M45-specific CD8$^+$ T cell populations across tissues from infant (10 DPN infection; pink) and adult (70 DPN infection; blue) mice. n = 10 mice per time-point per group. * p-value < 0.05 as assessed by two-way ANOVA with Bonferroni multiple comparisons test. Data are combined from N = 2 independent experiments. Tissue Abbreviations: blood (BLD), bone marrow (BOM), cervical lymph node (CLN), lung (LNG), liver (LVR), spleen (SPL), and salivary gland (SVG)

bioRxiv preprint doi: https://doi.org/10.64898/2026.03.16.712099; this version posted March 17, 2026. The copyright holder for this preprint (which was not certified by peer review) is the author/funder, who has granted bioRxiv a license to display the preprint in perpetuity. It is made available under aCC-BY 4.0 International license.

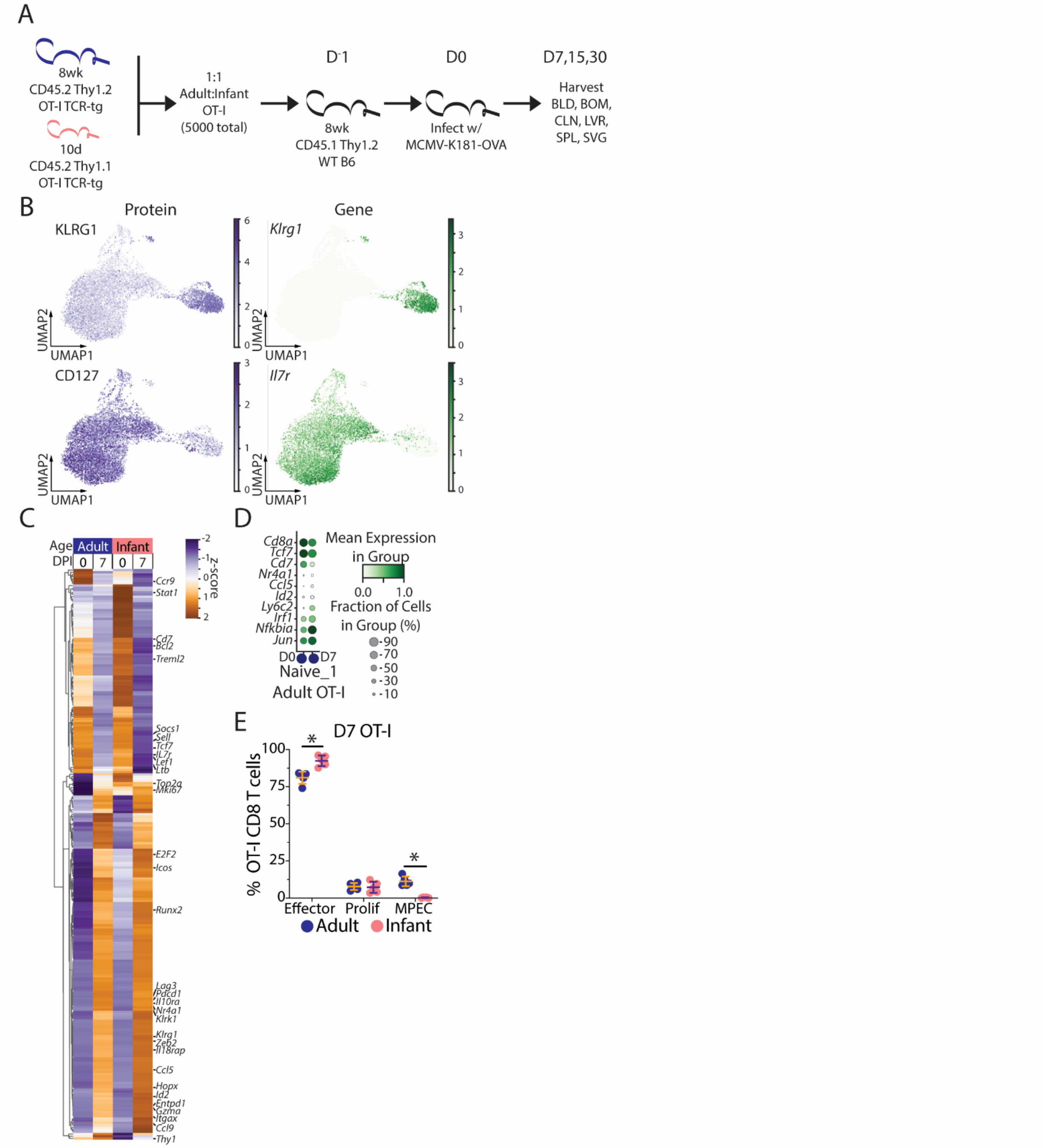


**Figure S5. Transcriptomic distinctions among infant and adult naive and MCMV-specific T cells in acute infection.**

(A) Experimental schematic for co-transfer of adult and infant TCR-transgenic OT-I CD8$^+$ T cells in Figure 4. (B) Expression of surface molecules bound by CITE-Seq antibodies (left) and corresponding gene expression (right) for KLRG1 (top) and CD127 (bottom). (C) Z-scored pseudobulk heatmap of the 100 most differentially expressed genes ($p<0.05$, $\log_2$(fold change)>1) from each group of enriched splenic OT-I CD8$^+$ T cells from adult (blue) and infant (pink) mice at 0 and 7 DPI. (D) Dot plot displaying the relative frequency and degree of expression for indicated genes in adult OT-I CD8$^+$ T cells in Naïve cluster 1 prior to (D0) and 7 days post-infection (D7). (E) Frequency of OT-I CD8$^+$ T cells classified as effector, proliferating, or MPEC from adult (blue) and infant (pink) donors at 7 days post-infection. n = 5 recipients *$p<0.05$ assessed by two-way ANOVA with Bonferroni multiple comparisons test.

bioRxiv preprint doi: https://doi.org/10.64898/2026.03.16.712099; this version posted March 17, 2026. The copyright holder for this preprint (which was not certified by peer review) is the author/funder, who has granted bioRxiv a license to display the preprint in perpetuity. It is made available under aCC-BY 4.0 International license.

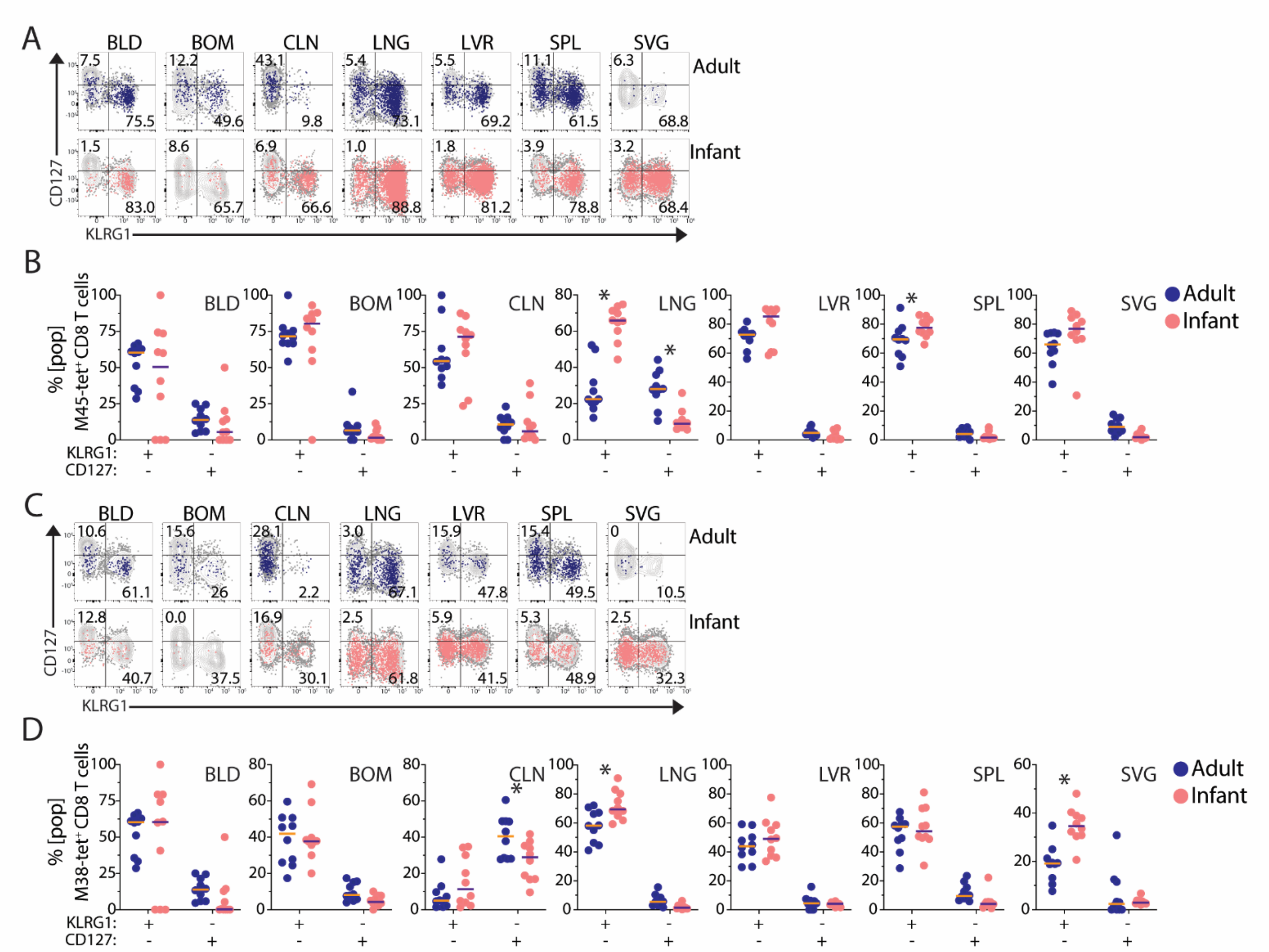


**Figure S6. Memory and effector potential of MCMV-specific T cell populations.**
Frequency of CD127⁻ KLRG1⁺ (SLEC) or CD127⁺ KLRG1⁻ (MPEC) of adult (blue) or infant (pink) M45-tetramer⁺ cells at 7 DPI overlaid on total CD8 T cells (grey) shown in representative flow cytometry plots from indicated sites (A) and compiled data from n=10 mice per group (B). (C, D) Same as (A-B) for M38-tetramer⁺ CD8 T cells. *$p < 0.05$ assessed by two-way ANOVA with Bonferroni multiple comparisons test. Data are combined from N = 2 independent experiments. Tissue Abbreviations: blood (BLD), bone marrow (BOM), cervical lymph node (CLN), lung (LNG), liver (LVR), spleen (SPL), and salivary gland (SVG)

bioRxiv preprint doi: https://doi.org/10.64898/2026.03.16.712099; this version posted March 17, 2026. The copyright holder for this preprint (which was not certified by peer review) is the author/funder, who has granted bioRxiv a license to display the preprint in perpetuity. It is made available under aCC-BY 4.0 International license.

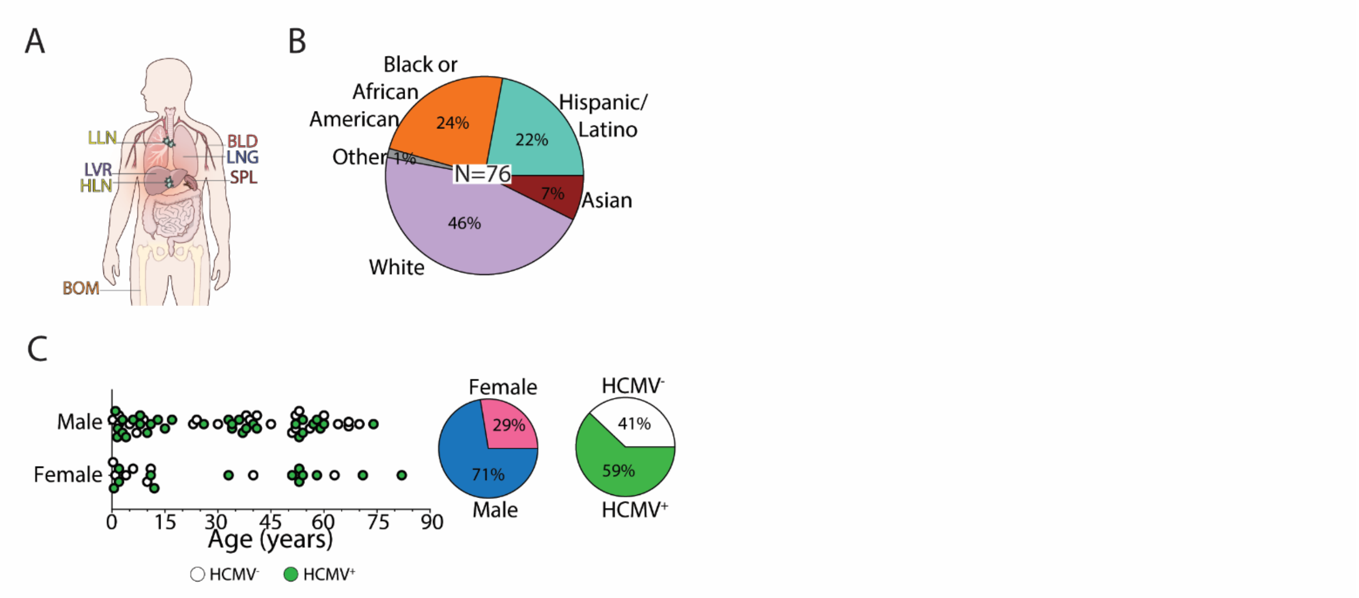


**Figure S7. Organ donor cohort demographics**

(A) Illustration of tissue sites analyzed by flow cytometry. (B) Reported ethnicities of the 76 organ donors utilized. (C) Distribution of HCMV-serostatus and Sex by Age. Tissue Abbreviations: blood (BLD), spleen (SPL), bone marrow (BOM), lymph nodes (LNs; hepatic and lung), liver (LVR), and lung (LNG)

bioRxiv preprint doi: https://doi.org/10.64898/2026.03.16.712099; this version posted March 17, 2026. The copyright holder for this preprint (which was not certified by peer review) is the author/funder, who has granted bioRxiv a license to display the preprint in perpetuity. It is made available under aCC-BY 4.0 International license.

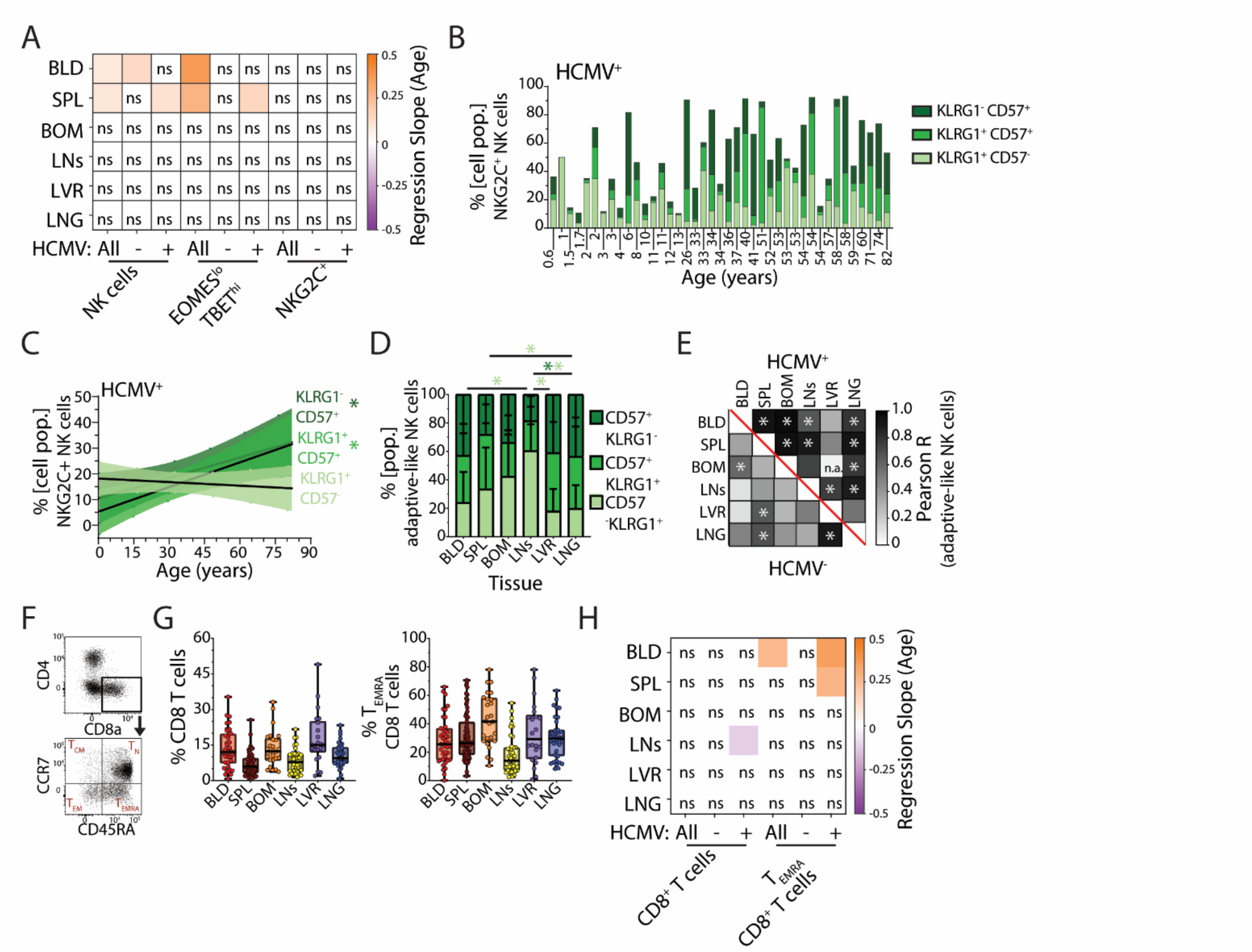


**Figure S8. Age and tissue influence adaptive-like NK cell and CD8$^{+}$ T$_{EMRA}$ cell phenotypic composition.** (A) Heatmap of linear regression slopes for tissue NK cells (Figure 6I,J; Figure 7B) with significant effects of age. Orange and purple correspond to increasing and decreasing frequencies of indicated cell populations with age from all, HCMV$^{-}$, and HCMV$^{+}$ donors. (B) Composition of CD57/KLRG1 expression among NKG2C$^{+}$ NK cells in spleen of HCMV$^{+}$ donors. (C) Linear regression for CD57/KLRG1 populations among NKG2C$^{+}$ NK cells by donor age in spleen of HCMV$^{+}$ donors. Colored bands correspond to confidence intervals. *$p < 0.05$ (D) Frequency of KLRG1/CD57 among adaptive-like ([KLRG1$^{+}$ or CD57$^{+}$] NKG2C$^{+}$) NK cells in tissues from HCMV$^{+}$ donors. *$p < 0.05$ assessed by two-way ANOVA with Bonferroni multiple comparisons test and pairing tissues within donors. (E) Pearson R correlation of the frequency of adaptive-like NK cells across tissues from HCMV$^{+}$ (top/ right) and HCMV$^{-}$ (bottom/ left) donors. (F) Representative gating of CD8 T cells and CD8 T$_{EMRA}$ cells. (G) Frequency of CD8$^{+}$ T cells among lymphocytes (left) and T$_{EMRA}$ among CD8$^{+}$ T cells (right) in indicated donor tissues. (H) Heatmap of linear regression slopes for tissue CD8 T cells (panel G; Figure 7F) with significant effects of age. Orange and purple correspond to increasing and decreasing frequencies of indicated cell populations with age from all, HCMV$^{-}$, and HCMV$^{+}$ donors. *$p < 0.05$. Tissue Abbreviations: blood (BLD), spleen (SPL), bone marrow (BOM), lymph nodes (LNs; hepatic and lung), liver (LVR), and lung (LNG)

bioRxiv preprint doi: https://doi.org/10.64898/2026.03.16.712099; this version posted March 17, 2026. The copyright holder for this preprint (which was not certified by peer review) is the author/funder, who has granted bioRxiv a license to display the preprint in perpetuity. It is made available under aCC-BY 4.0 International license.

**TABLE S1. Adult and Infant NK cell differential gene expression at 0,7,15 DPI**

**TABLE S2. Adult and Infant OT-I CD8 T cell differential gene expression at 0,7 DPI**

**TABLE S3. Gene Rank of Adaptive-like NK cell scHPF signature**

**TABLE S4. Donor Information Table**

| Donor # | Age (years) | Sex | HCMV serostatus |
|---|---|---|---|
| HDL093 | 0.58 | FEMALE | positive |
| LI014 | 1.00 | MALE | positive |
| LI012 | 1.50 | MALE | positive |
| LI015 | 1.666666667 | MALE | positive |
| HDL115 | 2 | FEMALE | positive |
| HDL138 | 2 | FEMALE | positive |
| HDL111 | 3 | MALE | positive |
| HDL133 | 3 | MALE | positive |
| HDL099 | 4 | MALE | positive |
| HDL136 | 6 | MALE | positive |
| D0582 | 8 | MALE | positive |
| LI003 | 8 | MALE | positive |
| D0609 | 10 | MALE | positive |
| D0658 | 11 | FEMALE | positive |
| HDL094 | 11 | MALE | positive |
| LI013 | 12 | FEMALE | positive |
| HDL114 | 13 | MALE | positive |
| D0624 | 15 | MALE | positive |
| D0717 | 17 | MALE | positive |
| D0604 | 26 | MALE | positive |
| D0516 | 33 | FEMALE | positive |
| D0646 | 33 | MALE | positive |
| D0420 | 34 | MALE | positive |
| D0487 | 34 | MALE | positive |
| D0634 | 36 | MALE | positive |
| D0627 | 37 | MALE | positive |
| D0577 | 38 | MALE | positive |
| D0457 | 40 | MALE | positive |
| D0629 | 41 | MALE | positive |
| D0510 | 51 | FEMALE | positive |
| D0630 | 52 | MALE | positive |
| D0354 | 53 | FEMALE | positive |
| D0541 | 53 | FEMALE | positive |
| D0542 | 53 | MALE | positive |
| D0554 | 54 | FEMALE | positive |
| D0556 | 54 | MALE | positive |
| D0615 | 54 | MALE | positive |
| D0544 | 57 | MALE | positive |
| D0612 | 58 | FEMALE | positive |
| D0625 | 58 | MALE | positive |

bioRxiv preprint doi: https://doi.org/10.64898/2026.03.16.712099; this version posted March 17, 2026. The copyright holder for this preprint (which was not certified by peer review) is the author/funder, who has granted bioRxiv a license to display the preprint in perpetuity. It is made available under aCC-BY 4.0 International license.

| | | | |
|---|---|---|---|
| D0689 | 59 | MALE | positive |
| D0553 | 60 | MALE | positive |
| D0574 | 71 | FEMALE | positive |
| D0691 | 74 | MALE | positive |
| D0540 | 82 | FEMALE | positive |
| LI004 | 0.25 | MALE | negative |
| LI008 | 0.33 | FEMALE | negative |
| LI018 | 1 | FEMALE | negative |
| HDL035 | 2 | MALE | negative |
| LI005 | 2 | MALE | negative |
| HDL123 | 4 | FEMALE | negative |
| HDL072 | 5 | MALE | negative |
| D0539 | 6 | FEMALE | negative |
| HDL150 | 7 | MALE | negative |
| HDL143 | 9 | MALE | negative |
| HDL128 | 10 | FEMALE | negative |
| HDL152 | 11 | FEMALE | negative |
| D0697 | 23 | MALE | negative |
| D0685 | 24 | MALE | negative |
| D0594 | 30 | MALE | negative |
| D0593 | 38 | MALE | negative |
| D0693 | 39 | MALE | negative |
| D0566 | 40 | FEMALE | negative |
| D0565 | 41 | MALE | negative |
| D0661 | 45 | MALE | negative |
| D0671 | 51 | MALE | negative |
| D0546 | 52 | MALE | negative |
| D0713 | 52 | MALE | negative |
| D0561 | 53 | MALE | negative |
| D0572 | 56 | MALE | negative |
| D0667 | 60 | MALE | negative |
| D0694 | 63 | FEMALE | negative |
| D0632 | 64 | MALE | negative |
| D0547 | 67 | MALE | negative |
| D0548 | 67 | MALE | negative |
| D0584 | 70 | MALE | negative |

**TABLE S5. Key resources used in this study**

| **Reagent or Resource** | **Source** | **Identifier** |
|---|---|---|
| Antibodies | | |
| Flow Cytometry | | |
| Anti-mouse CD11a BUV496 | BD | M17/4, Cat. #741071 |
| Anti-mouse CD11a PE-Cy7 | Biolegend | M17/4, Cat. #101122 |
| Anti-mouse CD127 eFlour 450 | ThermoFisher | eBioSB/199 (SB/199), Cat. #48-1273-82 |
| Anti-mouse CD14 FITC | Biolegend | Sa14-2, Cat. #123308 |
| Anti-mouse CD19 FITC | Biolegend | 6D5, Cat. #115506 |
| Anti-mouse CD3e AF700 | Biolegend | 500A2, Cat. #152316 |

bioRxiv preprint doi: https://doi.org/10.64898/2026.03.16.712099; this version posted March 17, 2026. The copyright holder for this preprint (which was not certified by peer review) is the author/funder, who has granted bioRxiv a license to display the preprint in perpetuity. It is made available under aCC-BY 4.0 International license.

| | | |
|---|---|---|
| Anti-mouse CD3e PerCP-Cy5.5 | Biolegend | 500A2, Cat. #152312 |
| Anti-mouse CD4 BV605 | Biolegend | GK1.5, Cat. #100451 |
| Anti-mouse CD4 PE-Cy5 | Biolegend | RM4-5, Cat. #100513 |
| Anti-mouse CD44 BUV805 | BD | IM7, Cat. #741921 |
| Anti-mouse CD44 BV605 | Biolegend | IM7, Cat. #103047 |
| Anti-mouse CD45 APC-Fire 810 | Biolegend | 30-F11, Cat. #103174 |
| Anti-mouse CD45 BUV395 | BD | I3/2.3, Cat. #567451 |
| Anti-mouse CD45.1 APC-Fire 810 | Biolegend | A20, Cat. #110703 |
| Anti-mouse CD45.2 BV421 | Biolegend | 104, Cat. #109832 |
| Anti-mouse CD5 PerCP-Vio 700 REAfinity | Miltenyi Biotec | REA421, Cat. #130-128-069 |
| Anti-mouse CD8a BV510 | Biolegend | 53-6.7, Cat. #100752 |
| Anti-mouse CD8a BV570 | Biolegend | 53-6.7, Cat. #100740 |
| Anti-mouse CD90.1 (Thy1.1) Pacific Blue | Biolegend | OX-7, Cat. #202521 |
| Anti-rat CD90/mouse CD90.1 (Thy1.1) PE | Biolegend | OX-7, Cat. #202524 |
| Anti-mouse CD90.2 (Thy1.2) APC | Biolegend | 53-2.1, Cat. #140312 |
| Anti-mouse CD90.2 (Thy1.2) PercP-Cy5.5 | Biolegend | 30-H12, Cat. #105337 |
| Anti-mouse KLRG1 PE-Cy7 | Biolegend | 2F1/KLRG1, Cat. #138416 |
| Anti-mouse Ly49H AF647 | Biolegend | 3D10, Cat. #144710 |
| Anti-mouse Ly6G FITC | Biolegend | 1A8, Cat. #127606 |
| Anti-mouse M38 APC | NIH Tetramer Core | H-2K(b) MCMV m38 316-323 SSPPMFRV |
| Anti-mouse M45 PE | NIH Tetramer Core | H-2D(b) MCMV m45 985-993 HGIRNASFI |
| Anti-mouse NK1.1 PE-CF594 | BD | PK136, Cat. #562864 |
| Anti-mouse TCR Vα2 AF488 | Biolegend | B20.1, Cat. #127820 |
| Anti-mouse TCR Vβ5.1/2 APC | Biolegend | MR904, Cat. #139506 |
| Anti-human Eomes BUV395 | BD | X4-83, Cat. #567171 |
| Anti-human CD94 BUV496 | BD | HP-3D9, Cat. #750232 |
| Anti-human CD14 BUV615 | BD | M5E2, Cat. #751150 |
| Anti-human CD19 BUV615 | BD | SJ254C1, Cat. #612989 |
| Anti-human NKG2C BUV805 | BD | 134591, Cat. #749683 |
| Anti-human Tbet BV421 | Biolegend | 4B10, Cat. #644816 |
| Anti-human CD5 Pacific Blue | Biolegend | UCHT2, Cat. # 300624 |
| Anti-human CD57 BV510 | Biolegend | QA17A04, Cat. #393314 |
| Anti-human CD8a BV570 | Biolegend | RPA-T8, Cat. #301038 |
| Anti-human CD4 BV605 | Biolegend | RPA-T4, Cat. #300556 |
| Anti-human CD45RA BV711 | Biolegend | HI100, Cat. #304138 |
| Anti-human CD127 AF532 | Thermo-Fisher | EBioRDR5, Cat. #58-1278-42 |
| Anti-human CD3 NovaFluor Blue 610-70S | Thermo-Fisher | UCHT2, Cat. #H002T03B06-A |
| Anti-human CD56 PE-CF594 | BD | NCAM16.2, Cat. #564849 |

bioRxiv preprint doi: https://doi.org/10.64898/2026.03.16.712099; this version posted March 17, 2026. The copyright holder for this preprint (which was not certified by peer review) is the author/funder, who has granted bioRxiv a license to display the preprint in perpetuity. It is made available under aCC-BY 4.0 International license.

| | | |
|---|---|---|
| Anti-human CD16 PE-Cy5 | Biolegend | 3G8, Cat. #302010 |
| Anti-human KLRG1 PE-Cy7 | Biolegend | SA231A2, Cat. #367720 |
| Anti-human CCR7 APC-R700 | BD | 3D12, Cat. #750232 |
| Anti-human CD45 APC-Fire810 | Biolegend | HI30, Cat. #304076 |
| | | |
| Enrichment kits | | |
| Dead cell removal kit | Miltenyi Biotec | Cat. #130-090-101 |
| NK cell Isolation Kit, mouse | Miltenyi Biotec | Cat. #130-115-818 |
| EasySep™ Mouse $CD8^+$ T cell Isolation Kit | STEMCELL Technologies | Cat. #19853 |
| | | |
| CITE-Seq Antibodies | | |
| TotalSeq-C0002 anti-mouse CD8a | Biolegend | 53-6.7, Cat. #100785 |
| TotalSeq-C0157 anti-mouse CD45.2 | Biolegend | 104, Cat. #109855 |
| TotalSeq-C0178 anti-mouse CD45.1 | Biolegend | A20, Cat. #11757 |
| TotalSeq-C0001 anti-mouse CD4 | Biolegend | RM4-5, Cat. #100571 |
| TotalSeq-C0075 anti-mouse CD90.2 | Biolegend | 30-H12, Cat. #105353 |
| TotalSeq-C0380 anti-mouse CD90.1 | Biolegend | OX-7, Cat. #202551 |
| TotalSeq-C0198 anti-mouse CD127 | Biolegend | A7R34, Cat. #135047 |
| TotalSeq-C0002 anti-mouse/human KLRG1 | Biolegend | 2F1/KLRG1, Cat. #138433 |
| TotalSeq-C0301 anti-mouse Hashtag 1 | Biolegend | Cat. #155861 |
| TotalSeq-C0302 anti-mouse Hashtag 2 | Biolegend | Cat. #155863 |
| TotalSeq-C0303 anti-mouse Hashtag 3 | Biolegend | Cat. #155865 |
| TotalSeq-C0304 anti-mouse Hashtag 4 | Biolegend | Cat. #155867 |
| TotalSeq-C0305 anti-mouse Hashtag 5 | Biolegend | Cat. #155869 |
| TotalSeq-C Mouse Universal Cocktail, V1.0 | Biolegend | Cat. #199903 |
| | | |
| Cell Depletions | | |
| InVivoMAb Anti-mouse CD3e depleting antibody 145-2C11 | BioXcell | Cat. #BE0001-1 |
| InVivoMAb Anti-mouse NK1.1 depleting antibody PK136 | BioXcell | Cat. #BE0036 |
| InVivoMAb Armenian Hamster IgG Isotype PIP | BioXcell | Cat. #BE0260 |

bioRxiv preprint doi: https://doi.org/10.64898/2026.03.16.712099; this version posted March 17, 2026. The copyright holder for this preprint (which was not certified by peer review) is the author/funder, who has granted bioRxiv a license to display the preprint in perpetuity. It is made available under aCC-BY 4.0 International license.

| | | |
|---|---|---|
| InVivoMAb Mouse IgG2a Isotype C1.18.4 | BioXcell | Cat. #BE0085 |
| | | |
| Chemicals and Reagents | | |
| ACK Lysing buffer | Gibco | Ref. #A10492-01 |
| Buffer ATL | Qiagen | Cat. #939011 |
| Collagenase | Millipore Sigma | Cat. #11088882001 |
| DNAse | Millipore Sigma | Cat. #DN25-5G |
| DNeasy Blood & Tissue Kit (250) | Qiagen | Cat. #69506 |
| DPBS | Corning | Cat. #20-030-CV |
| EDTA | Corning | Cat. #46-034-CI |
| Fetal Bovine Serum | GeminiBio | Cat. #100-106 |
| Fixable Viability Dye Zombie NIR | Biolegend | Cat. #423106 |
| Fixation Buffer | Invitrogen | Cat. #00-5223-56 |
| Foxp3 / Transcription Factor Fix/Perm Diluent (1X) | FisherScientific | Ref. #TNB-1022-L160 |
| IMDM | Gibco | Ref#122440-053 |
| Ionomycin | Sigma | Cat#I9657 |
| Penicillin/Streptomycin/L-glutamine | GeminiBio | Cat#c400-110 |
| Proteinase K | Qiagen | Cat. #19133 |
| RPMI 1640 | Corning | Cat. #10-040-CM |
| Solution 13 AO/DAPI Staining Reagent | Chemometec | Cat. # 910-3013 |
| SsoAdvanced Universal SYBR Green Supermix® | BioRad | Cat. #1725274 |
| Tonbo Flow Cytometry Perm Buffer (10X) | FisherScientific | Ref. #TNB-1213-L150 |
| Tonbo Foxp3 / Transcription Factor Fix/Perm Concentrate (4X) | FisherScientific | Ref. #TNB-1020-L050 |
| TruStain FcX PLUS (anti-mouse CD16/32) | Biolegend | Cat. #156604 |
| | | |
| Software and Algorithms | | |
| FlowJo™ v 10.9 software | BD | https://www.flowjo.com |
| SpectroFlo® 3.1.0 software | CytekBio | https://www.cytekbio.com/pages/spectro-flo |
| Prism v 10.6 software | Graphpad | https://www.graphpad.com |
| BR.io cloud platform | BioRad | https://www.br.io |
